# Evaluation of Anti-inflammatory and Antimicrobial Properties of Mustard Seed Extract-Based Hydrogel: An In Vitro Study

**DOI:** 10.7759/cureus.45146

**Published:** 2023-09-13

**Authors:** Devika Bajpai, Sankari Malaiappan, Rajeshkumar S

**Affiliations:** 1 Periodontology, Saveetha Institute of Medical and Technical Sciences, Chennai, IND; 2 Pharmacology, Saveetha Institute of Medical and Technical Sciences, Chennai, IND

**Keywords:** innovative gel, local drug delivery, periodontitis, anti-microbial, anti-inflammatory, mustard seed extract

## Abstract

Introduction: Mustard has been regarded as one of the world's most extensively produced and useful plants as well as one of the oldest condiments ever. The aim of the study was to develop and analyse the anti-inflammatory and antimicrobial properties of mustard seed extract.

Methods: The extract was prepared by using a double filtration technique and anti-inflammatory properties were checked using egg albumin assay and bovine serum albumin assay and diclofenac sodium was the control. The antimicrobial property was evaluated by the Kirby-Bauer test and chlorhexidine gel was the control. The species included were *Staphylococcus aureus*, *Streptococcus mutans*, *Enterococcus faecalis* and *Candida albicans*.

Results: The results showed that the anti-inflammatory property of mustard seed extract is comparable to diclofenac sodium whereas the maximum zone of inhibition was seen against *C. albicans*.

Conclusion: This study discovered that mustard seed extract has potent antimicrobial and anti-inflammatory activity against a variety of oral microorganisms. These findings indicate that this hydrogel was highly active against the tested pathogens and will be effective in the treatment of periodontitis.

## Introduction

Chronic periodontitis is a common and progressive form of gum disease (periodontal disease) that affects the supporting structures of the teeth, including the gingiva, periodontal ligament, and alveolar bone. It is characterized by inflammation of the periodontal tissues and destruction of the supporting structures over time [1].

Treatment of periodontitis involves non-surgical or surgical management with adjunctive use of chemotherapeutic agents. The only disadvantage of using chemotherapeutic agents is the adverse effects caused by these agents. To avoid this, various natural or herbal extracts are being used to minimise the side effects and enhance therapeutic levels of the drug locally at the affected site. Therefore, local drug delivery (LDD) involves placing a drug or therapeutic substance directly at the target site, which can enhance the efficacy of treatment while minimizing systemic side effects. Herbal extracts are often encapsulated in various delivery systems, such as gels, creams, nanoparticles, or hydrogels, to ensure controlled release and sustained therapeutic effects and therefore it is the rationale behind the development of such extracts [2].

For thousands of years, mustard has been regarded as one of the world's most extensively produced and useful plants as well as one of the oldest condiments ever. The earliest mustard plant cultivars and uses are documented from 3000 B.C. [3]. There are about 330 genera and over 3700 species of mustard plants, together referred to as Brassicaceae, which are members of the order Brassicales (formerly known as Capparales) [4]. Black mustard (Brassica nigra) is planted extensively. It has black dark-red seeds that are more pungent as well as bitter than those of white (Sinapis alba) or brown (Brassica juncea) mustard. Black mustard seeds contain a significant amount of sinigrin, a glucosinolate that can be hydrolyzed to allyl-isothiocyanate (AITC), which has a peculiar, strong, unpleasant odour [5]. Although black mustard is frequently in use as a spice, herb, and oil source, challenges with harvesting it in the United States (U.S.) and Europe have a negative impact on its popularity [6]. Mustard seeds have been used for culinary purposes since the beginning of time [7]. Mustard seeds contain bioactive molecule isothiocyanates (ITCs). In a study, *Pseudomonas aeruginosa* has demonstrated the activity of ITCs against biofilms [8]. Long ago, the mustard plant was more commonly used for medicinal purposes but later it became a popular ingredient as a spice. The medicinal properties were due to the presence of biologically active compounds in the seeds. This "bioactive compound" is present in a variety of sources with distinct functions.

The aim of this in-vitro study was to assess the anti-inflammatory and antimicrobial role of mustard seed extract on *Staphylococcus aureus*, *Streptococcus mutans*, *Enteroco faecalis* and *Candida albicans*.

## Materials and methods

Preparation of the hydrogel

**Figure 1:**
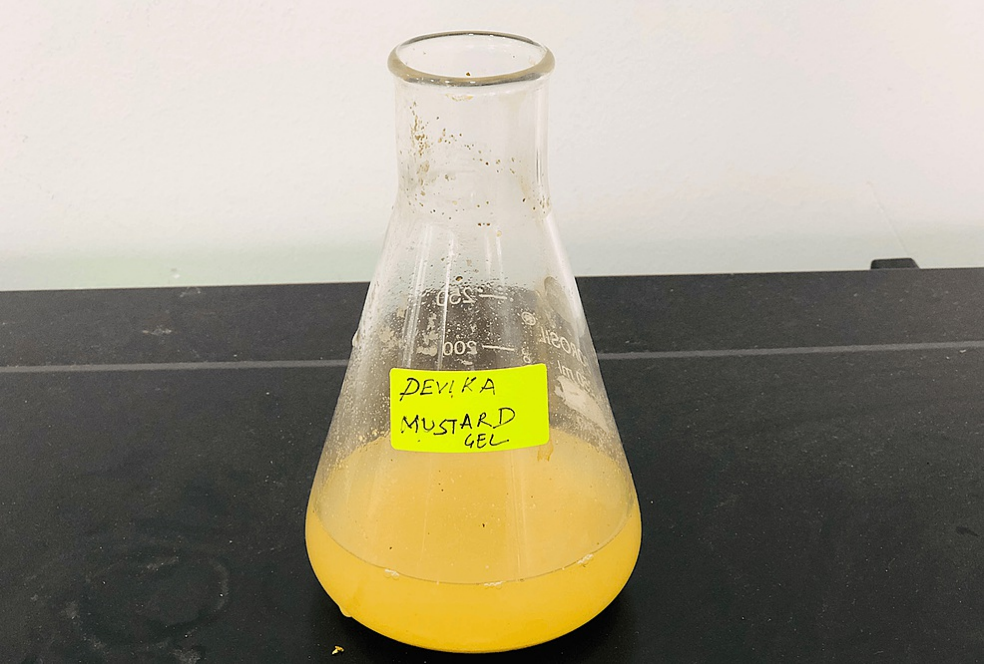
Preparation of mustard seed extract Preparation of mustard seed extract-based hydrogel was done by keeping the extract on the heater for 40 min at 65°C.

**Figure 2:**
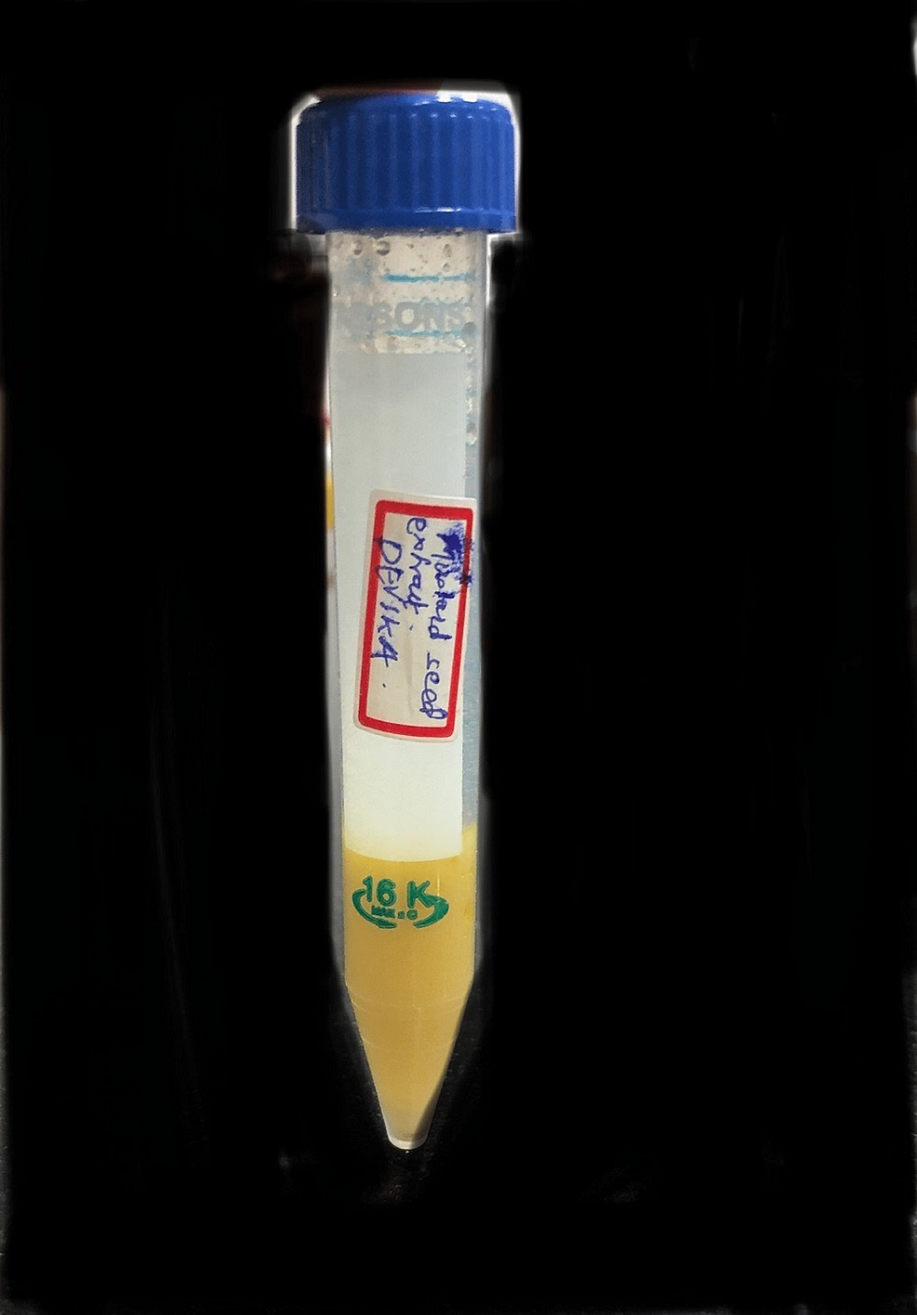
Mustard seed extract hydrogel Mustard seeds extract-based hydrogel made by double filtration method.

Mustard seed extract-based hydrogel was prepared by using 3 g of mustard seed powder in 100 ml of deionised (DI) water and was boiled at 65 degrees Celsius for 4 hours. The supernatant was discarded and the pellet was double-filtered. This process was repeated twice and the mustard seed extract was obtained. Carbopol (50 gm) was added to make hydrogel (Figures 1, 2).

Anti-inflammatory test

Albumin Denaturation Assay

The assay was done to evaluate the anti-inflammatory action of mustard seed hydrogel using bovine serum albumin (BSA) as a model protein. The denaturation of BSA was measured by its absorbance at 660 nm after treatment with mustard seed extract hydrogel at different concentrations. Mustard seed extract hydrogel was prepared in various concentrations. A total of 0.05 mL of the different concentrations of mustard seed extract hydrogel was mixed with 0.45 mL of 1% aqueous BSA solution. This solution was kept slightly acidic with a pH of 6.3. To achieve this pH, 1N hydrochloric acid (HCl) was used. The prepared samples were incubated at room temperature for 20 minutes. Before incubation, samples were preheated at 55°C for 30 minutes followed by cooling to room temperature. Diclofenac sodium is used as a standard control, which likely has known anti-inflammatory properties.

Protein Denaturation Assay

A solution measuring 5 ml was prepared by combining 2.8 ml of phosphate-buffered saline (PBS) with a pH of 6.3 and 0.2 ml of egg albumin (EA) extract prepared from hen eggs. For mustard seed extract, specific concentrations were prepared separately as (10 L, 20 L, 30 L, 40 L, 50 L). The positive control was diclofenac sodium. All the prepared solutions were heated in a 37°C water bath for 15 minutes followed by cooling down of the samples to room temperature and then absorption at 660 nm was measured.

Antimicrobial test

**Figure 3:**
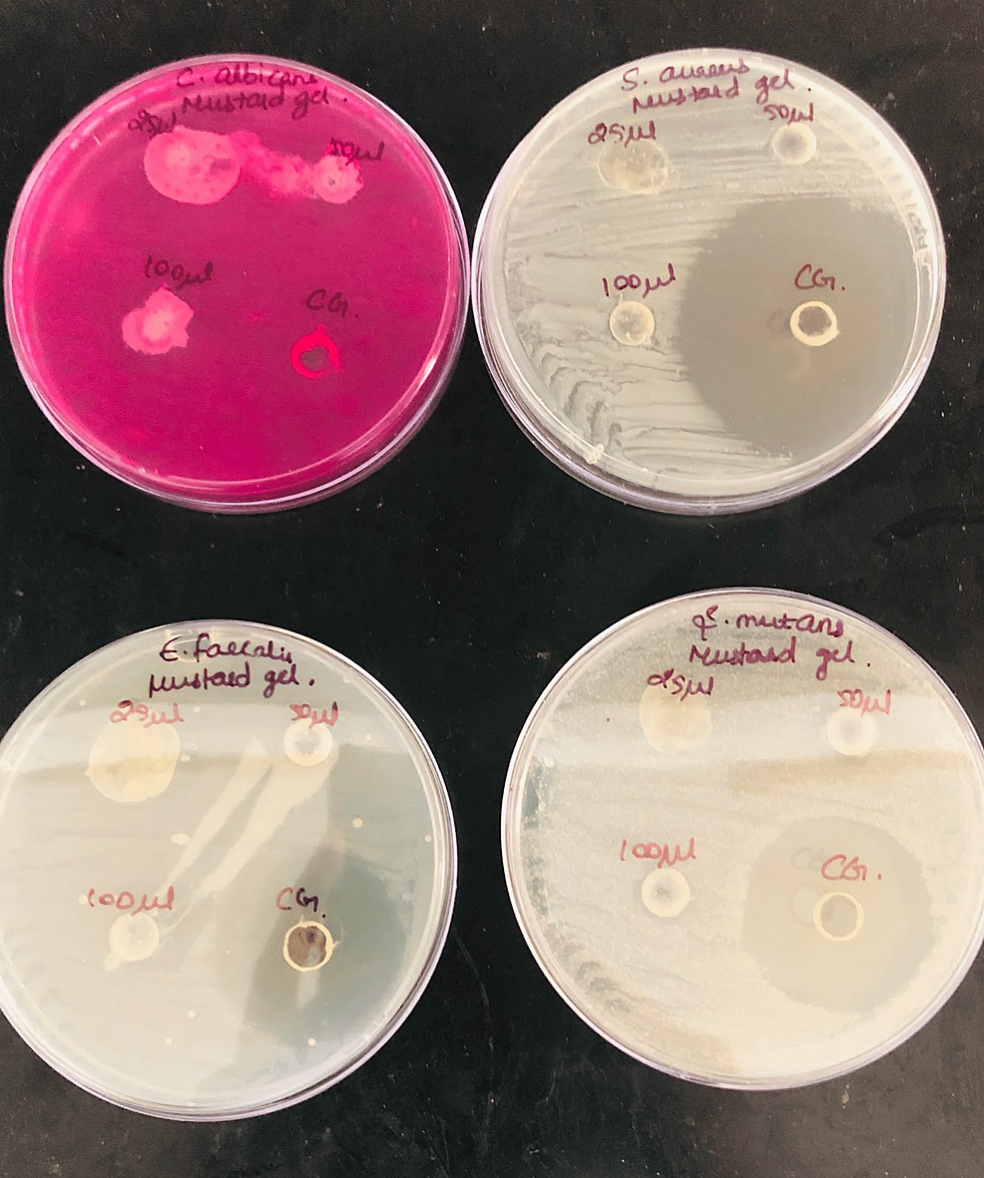
Cell culture test for antimicrobial action The antimicrobial test was performed on *S. mutans, S. aureus, C. albicans, E. faecalis*.

The Zone of Inhibition test (Kirby-Bauer Test) was used to determine antimicrobial activity. A nutrient agar-coated petri dish was streaked with the required bacteria culture (*S. aureus, C. albicans, E. faecalis, S. mutants*). A piece of mustard seed extract hydrogel 3cmx3cm was cut and placed on the nutrient agar and was incubated in a petri dish for 18-24 hours at 36°C so as to allow microbial growth in the culture medium (Figure 3).

The growth of bacteria on culture plates was observed and was seen as a dense yellow lawn after the incubation period. A clearance zone was visible on the test pieces. The size of this zone was measured.

## Results

Anti-inflammatory test

**Figure 4:**
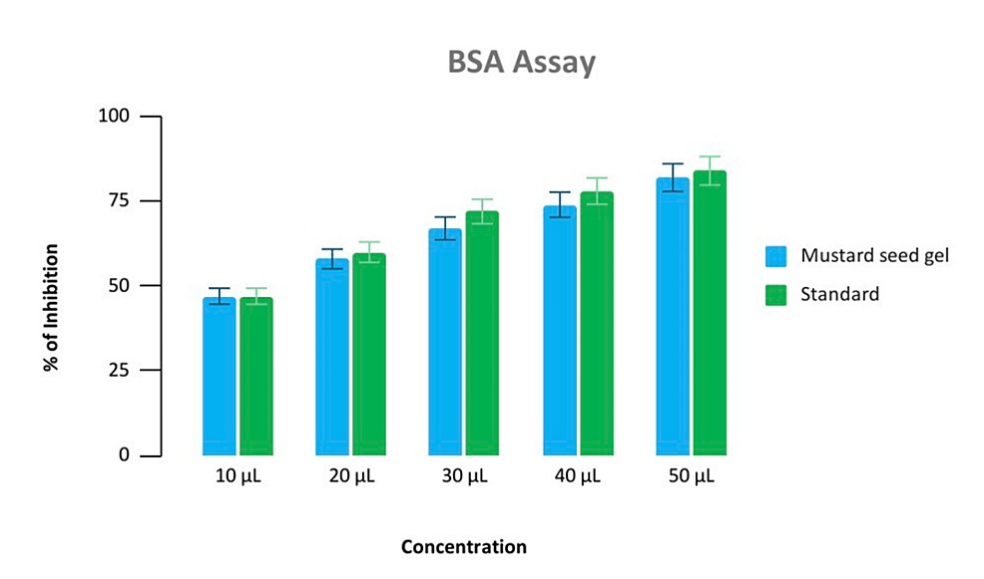
Anti-inflammatory test Percentage of inhibition test of mustard seed extract and diclofenac sodium in BSA (bovine serum albumin assay) at different concentrations in microlitre (10 μL, 20 μL, 30 μL, 40 μL, 50 μL).

**Figure 5:**
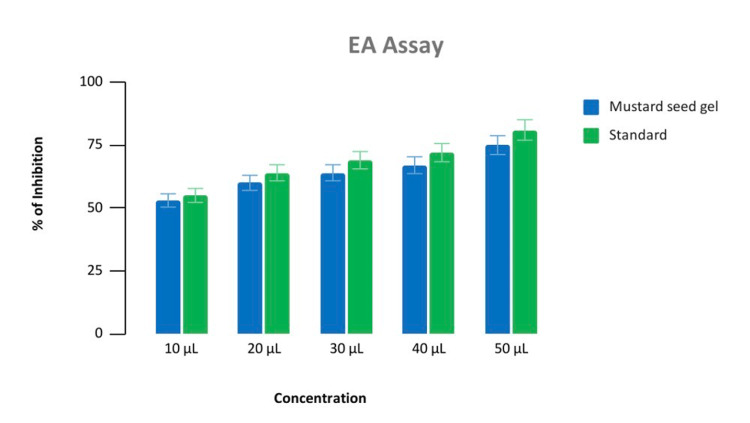
Anti-inflammatory test Percentage of inhibition test of mustard seed extract and diclofenac sodium in EA (egg albumin assay) at different concentrations in microlitre (10 μL, 20 μL, 30 μL, 40 μL, 50 μL).

In the BSA test the percentage of inhibition was similar at low concentrations i.e., 10 microlitres. In higher concentrations, the anti-inflammatory effect of the hydrogel was comparable with the standard i.e., diclofenac sodium whereas in the EA test, it was similar to the standard in all the concentrations (Figures 4, 5).

Antimicrobial test

The zone of inhibition for each of the above-mentioned microorganisms was tested. Zone of inhibition was observed for all the species and it was effective against *C. albicans* at 100 microlitre concentration whereas for other microorganisms it was lesser as compared to the commercial gel (Table 1). This illustrates that the gel could be effective against candidal infections also.

**Table 1:** Antimicrobial test Zone of inhibition test was measured in millimetres at different concentrations (in microlitres) of mustard seed extract.

Organism	Zone of inhibition (in mm)	Zone of inhibition (in mm)	Zone of inhibition (in mm)	Zone of inhibition (in mm)
	at 25ul conc.	at 50ul conc.	at 100ul conc.	CG (commercial gel)
S. aureus	24	24	38	40
C. albicans	9	9	16	12
E. faecalis	18	18	32	36
S. mutans	21	21	31	35

## Discussion

The antimicrobial and anti-inflammatory properties of mustard seed extract against oral pathogenic bacteria have not been studied in detail, so far. Hence the aim of this study was to evaluate these properties in mustard seed extract-based hydrogel. The results of this study were in accordance with the previous studies done for phytotherapeutic agents.

The pathogens included in this in-vitro study were primary colonisers and the antimicrobial test results showed that these organisms were susceptible to the mustard seed extract-based hydrogel in higher concentrations but less equivalent to the commercial gel (chlorhexidine). However, when compared to the control, diclofenac sodium, the extract's anti-inflammatory efficacy was comparable. These findings support and extend previous findings by Conrad A et al. in which it has been shown that microorganisms related to endocarditis are prone to mustard oil-containing plants because of their antimicrobial action [9]. Although chlorhexidine has been used as a benchmark against oral biofilm [10], it has been shown to have side effects in human gingival fibroblasts, osteosarcoma cells, and osteoblasts [11,12]. Furthermore, some of the salivary components can nullify the antimicrobial action of chlorhexidine against some oral pathogens [13]. In addition to it, a correlation between resistance to chlorhexidine as well as antibiotics by various microorganisms cannot be ruled out because of the mechanisms such as multidrug efflux pumps and changes in the cell membrane, which have been reported in certain reviews [14]. Listerine® [15] is another commonly used oral health product. Although there is mounting evidence that Listerine® improves oral health, there is a lack of evidence regarding antitoxic properties that precludes a precise safety evaluation [16].

Billions of microorganisms live in the oral cavity, and some of them are responsible for the development of various systemic diseases [17]. Oral health and overall health are linked [18]. As a result, maintaining good oral health is critical. Oil pulling was a traditional Ayurvedic treatment for the maintenance of oral health [19]. Oil pulling is easily accessible in the home [20]. Oil pulling is referred to as 'Kavala Graha' or 'Kavala Gandoosha' in the ayurvedic ancient literature [21]. Mustard oil is one of the oils used in oil pulling.

Mustard seeds have been grown and have been in use for a long time. Its role as a medicine, spice and cooking oil is well known. Because of their nutritional and functional properties, the seeds of the different mustard species are increasingly being used in the food and beverage industry. ITC is the biologically active compound present in the seeds that imparts a distinctive flavour and has potential health effects [22]. Many studies discovered possible side effects of oil pulling such as lipoid pneumonia caused by aspiration or inhalation of oily substances because this oil can be contaminated by oral pathogens and toxins, as well as stomach upset, nausea, and heavy metal poisoning caused by the presence of certain chemicals such as arsenic, lead, and mercury in ayurvedic preparations [23].

As a result, in cases of localised periodontal pockets, local delivery of the therapeutic agent is preferred over systemic administration because the high levels of the therapeutic agent are present at the site of infection thereby minimising the adverse effects caused by systemic drugs. The majority of systemic antimicrobials are linked to microbial resistance due to frequent use, poor availability and failure to achieve optimum concentration [24]. Because of these disadvantages, systemic administration of drugs has now been considered suitable for patients with widespread periodontal disease. These visible restraints have fueled a desire to begin periodontal treatment with LDDs. The therapeutic benefit of LDDs is accomplished by directly introducing the drug subgingivally into the pocket, which results in either sustained or controlled release of the biologically active compound to combat that exhibits its action against pathogenic microorganisms while minimising its systemic adverse effects [25]. So far, the antimicrobial effects of mustard seed extract on oral pathogenic bacteria have not been studied in depth. Therefore, this study aims to investigate the vulnerability of oral microorganisms of clinical importance and to demonstrate that mustard seed extract can be used as an LDD agent which can be used as an adjunct to scaling and root planing in patients with periodontitis.

As a result, this novel natural antimicrobial and anti-inflammatory hydrogel mustard seed extract can be a potentially effective component for oral health care. However, a comparison of this extract with periodontal pathogens is necessary to be done, and that is the limitation of this study approach. Another limitation of the study was mustard seeds have a peculiar pungent smell and that in future preparation should be masked by taste enhancers.

## Conclusions

This study discovered that mustard seed extract has potent antimicrobial activity against a variety of oral microorganisms. The extract has potent anti-inflammatory properties. These properties could potentially be harnessed in the form of a hydrogel to help treat periodontitis. Further animal studies need to be done followed by clinical studies to evaluate their cytotoxicity and effectiveness as LDD agents against various oral pathogens.
